# Screening of *Baccaurea ramiflora* (Lour.) extracts for cytotoxic, analgesic, anti-inflammatory, neuropharmacological and antidiarrheal activities

**DOI:** 10.1186/s12906-018-2100-5

**Published:** 2018-01-30

**Authors:** Mst. Luthfun Nesa, S. M. Sajedul Karim, Khairunasa Api, Md. Moklesur Rahman Sarker, Md. Monirul Islam, Asma Kabir, Mithun Kumar Sarker, Kamrun Nahar, Mohammad Asadujjaman, Mohammad Sirajum Munir

**Affiliations:** 1 Department of Pharmacy, Atish Dipankar University of Science and Technology, Dhaka, Bangladesh; 2 Department of Pharmacy, State University of Bangladesh, 77 Satmasjid Road, Dhaka, 1205 Bangladesh; 3 Department of Pharmacy, Northern University, Dhaka, Bangladesh; 4 Beximco Pharmaceutical Ltd, Dhaka, Bangladesh

**Keywords:** *Baccaurea ramiflora*, Cytotoxicity, Analgesic, Anti-inflammatory, CNS depressant, Antidiarrheal

## Abstract

**Background:**

It has been observed that the various part of *Baccaurea ramiflora* plant is used in rheumatoid arthritis, cellulitis, abscesses, constipation and injuries. This plant also has anticholinergic, hypolipidemic, hypoglycemic, antiviral, antioxidant, diuretic and cytotoxic activities. The present studyaimed to assess the cytotoxic, analgesic, anti-inflammatory, CNS depressant and antidiarrheal activities of methanol extract of *Baccaurea ramiflora* pulp and seeds in mice model.

**Methods:**

The cytotoxic activity was determined by brine shrimp lethality bioassay; anti-nociceptive activity was determined by acetic acid**-**induced writhing, formalin**-** induced licking and biting, and tail immersion methods. The anti**-**inflammatory, CNS depressant and anti-diarrheal activities were assessed by carrageenan**-**induced hind paw edema, the open field and hole cross tests, and castor oil**-**induced diarrheal methods, respectively. The data were analyzed by one way ANOVA (analysis of variance) followed by Dunnett’s test.

**Results:**

In brine shrimp lethality bioassay, the LC_50_ values of the methanol extracts of *Baccaurea ramiflora* pulp and seed were 40 μg/mL and 10 μg/mL, respectively. Our investigation showed that *Baccaurea ramiflora* pulp and seed extracts (200 mg/kg) inhibited acetic acid induced pain 67.51 and 66.08%, respectively (*p* < 0.05) that was strongly comparable with that of Ibuprofen (72%) (*p* < 0.05). The *Baccaurea ramiflora* pulp and seed extracts (200 mg/kg) significantly (*p* < 0.05) reduced 58.5 and 53.4 in early and 80.8%, 76.61% in late phase of formalin**-**induced licking and biting. At 60 and 90 min pulp and seed extracts (200 mg/kg) inhibited nociception of thermal stimulus 50.16 and 62.4%, respectively (*p* < 0.05) which was comparable with the standard (morphine, 75.9% inhibition). The pulp and seed extracts (200 mg/kg) significantly (*p* < 0.05) reduced inflammation (42.00 and 55.22%, respectively) in carrageenan**-**induced hind paw edema and defecations (59.7 and 63.03%, respectively) in castor oil induced diarrhea. Both the extracts showed high sedative activity at 30, 60, 90, and 120 min.

**Conclusion:**

Our investigation demonstrated significant cytotoxic, analgesic, anti-inflammatory, CNS depressant and antidiarrheal activities of methanol extract of *Baccaurea ramiflora* pulp and seeds (200 mg/kg).

## Background

Medicinal plants and natural medicines are a huge source of bioactive compounds that can be used for the discovery of new therapeutic compounds and the management of a wide range of diseases [1–3]. Many scientific reports showed the antidiabetic [4–6], anti-oxidant [7], immune stimulating [8–10], anti-inflammatory [11], antidiarrhoeal [7], anthelmintic [7], cytotoxic [12], and anti-obesity [13] activities of natural compounds or different herbal preparations. *Baccaurea ramiflora* (Lour. family of Euphorbiaceae) is a resourceful plant which has number of uses. The familiar names include Bhubi or Latkan (Bengali), Mafai (Thai), Leteku (Hindi) and Burmese grape. The slow-growing evergreen tree of *Baccaurea ramiflora (B. ramiflora*) has fruit (1–2″ around) and the fruit is yellow to red in color. This fruit tree is native to the Southeast Asian region and found growing wild in South China, Indo-China, India, Nepal, Myanmar, the Andaman Islands, Thailand and Peninsular Malaysia [14, 15].

The *B. ramiflora* is utilized as an antichloristic and anodyne against rheumatoid arthritis, abscesses, cellulitis, and treat injuries in Chinese Dai medicine [16]. The plant is also used as medicine by hill-tribes in Northern Thailand [17]. The fruit acts as antiviral and antioxidant and the stem bark acts as diuretic [18].

*B. ramiflora (*Lour) is such an underexploited fruit crop grown mainly in backyard plantation and as a forest plant. Research on *B. ramiflora* has been reported for its ethnobotanical uses, seed biology, and its isolated chemical constituents of essential oil. Three novel and four recognized compounds were isolated from the *Baccaurea ramiflora* stems [19]. The two new phenols, 6′- O-vanilloylisotachioside and 6′- O-vanilloyltachioside, together with nine known compounds, were isolated from the leaves of *B. ramiflora* (Euphorbiaceae) [17]. The rosmarinic acid that identified in *Baccaurea ramiflora* leaf can inhibit eicosanoids (e.g. prostaglandin biosynthesis) that is the final product of the cyclooxygenase pathway. Moreover, the phytochemical also can reduce the arachidonic acid level which indicates the antioxidant and anti-inflammatory activities of *B. ramiflora* [20]. The fractions of ethanol extracts of *Baccaurea ramiflora* (Lour.) leaves and stems showed potential cytotoxic activity [21].

*B. ramiflora* fruit is popular due to the high content of vitamin C, protein and iron. The plant parts are used to make wine and to treat abscesses, injuries and arthritis. They are also stewed [22]. The hydro methanol extract of the fruit pericarp of *B. ramiflora* showed significant DPPH scavenging activity [23]. These reviews clearly establish *B. ramiflora* as a medicinal plant which is underutilized and though commonly available but due to its less appealing nature and taste not gain much attention in civilized society. It tolerates unfavorable ecological condition and can be grown in unfertile lands.

These fruits have been used in folk medicine; quite a few of these are suitable for processed products. But most have not undergone any volitional stage of domestication and human selection. In animal models, phytochemical studies show various biochemical and pharmacological activities. The analysis showed that an appreciable amount of saponins and alkaloids remain in pulps (8.27 and 7.48%). The saponin containing fruits has anti-inflammatory activity [24]. The presence of alkaloids can also contribute for their analgesic, anti-apasmodic, and anti-bacterial properties [25].

Many researcher proved that flavonoids, phenolic compounds, tannins, alkaloids, saponins have analgesic, anti-inflammatory, antidiarrheal effect [25–28]. Therefore, the main objective was to assess the cytotoxic, anti-inflammatory, analgesic, CNS depressant and anti-diarrheal activities of methanol extracts of *Baccaurea ramiflora* pulp and seeds (MEBRP and MEBRS), respectively.

## Methods

### Plant material

The fresh fruits of *B. ramiflora* collected from the area of Rajshahi, Bangladesh. The plant was identified by a Taxonomist of Bangladesh National Herbarium, Dhaka, and a voucher specimen (38586) was retained there. Then pulp and seed were separated and dried for 1 week. Then dried plant part is pulverized into a coarse powder with a suitable grinder. The prepared powder was poured in an airtight container and placed in a cool, dark and dry place extraction.

### Preparation of extracts

The pulp and seed powdered materials were placed in a fresh, smooth bottomed glass container for soaking in 85% methanol. The container was preserved up to 7 days within frequent shaking and stirring. The whole mixture was filtered through a coarse filtration material (a piece of clean and white cotton) and then filtered with Whatsman filter paper (Bibby RE200, Sterilin Ltd., UK). The filtrates (methanol extract) were evaporated using rotary evaporator and looked like a gummy concentrate black color which referred to as crude methanol extract of pulp and seed. The resulting extracts were stored in a blocked container for protection and further use.

### Animals

The Swiss albino mice (male, 20-25 g) were taken from International Centre for Diarrheal Disease Research, Bangladesh (ICDDRB). Under ambient temperature all animals were kept with 12 h light followed by a 12 h dark cycle. Prior to actual experiments, the animals were acclimatized for 1 week. The animals are separated into six groups in which five mice present in each group. Experiments on animals were performed in accordance with guidelines of the Ethical Committee of Pharmacy Department, Atish Dipankar University of Science and Technology, Dhaka, Bangladesh.

### Chemicals

Ibuprofen, diazepam and loperamide were obtained from Beximco Pharmaceuticals Ltd., Bangladesh; Merck gave formalin and acetic acid, Germany. Bangladesh. BDH chemicals Ltd. provided Tween 80, normal saline water (0.9% NaCl), castor oil, carageenan and vincristine sulphate.

### Screening of cytotoxic activity

Brine shrimp lethality bioassay was determined by the method as described earlier [29]. In this suitable test simple zoological organism (*Artemiasalina*) was used for the screening. The brine shrimp eggs were taken from an aquarium shop (Dhaka, Bangladesh) and mature shrimp (called nauplii) hatched in artificial seawater (3.8% NaCl solution) for 48 h. The extracts was dissolved in DMSO (not more than 50 μL DMSO in 5 mL solution to avoid toxicity of itself) and sea water (3.8% NaCl in water) to prepare 10, 20, 40, 80 and 160 μg/mL concentration respectively. 50 μL DMSO was diluted to 5 mL for control group. Then mature shrimps were added to each all experimental and control vials. After 24 h the numbers of the dead nauplii were counted. After 24 h the LC_50_ (median lethal concentration) of the sample was calculated by a plot of percentage the dead shrimps against the logarithm of the sample concentration. Vincristine sulphate was used as a reference standard.

### Determination of analgesic activity by acetic acid-induced writhing method

The acetic acid-induced writhing model was used to determine analgesic activity [30]. Test samples MEBRP, MEBRS (100 and 200 mg/kg body weight respectively), vehicle (1% tween 80 in water) and positive control (Ibuprofen, 10 mg/kg p.o.) were given and after 30 min 0.1% acetic acid was injected intra-peritoneally. The writhing (specific contractions of body) were observed randomly and its frequency was counted for up to 25 min in each group of animals [31]. Sometimes the animals showed contraction but they did not complete it which was considered as half writhing. Accordingly, two half-writhing were counted as one full writhing. The number of writhes in each sample group was compared to control group.

The percent inhibition (% analgesic activity) was calculated by

$$ \%\mathrm{inhibition}=\left\{\left(\mathrm{A}\hbox{-} \mathrm{B}\right)/\mathrm{A}\times 100\right. $$

Where, A = Average number of writhing of the control group; B = Average number of writhing of the test group.

### Determination of analgesic activity by formalin test

The formalin test is used to determine the analgesic activity [31]. The MEBRP, MEBRS (100, 200 mg/kg, p.o. respectively) and Ibuprofen (10 mg/kg, p.o.) were orally administered and after 30 min 20 μL of 5% formalin was injected into the dorsal surface of the right hind paw in each group. The number of licking and biting was counted up to 30 min. The early phase time was 5 min and the late phase time was 15 to 30 min of post formalin injection. The total number of licking and biting (pain behavior) of the injured paw was calculated with a stop watch.

### Determination of analgesic activity by tail immersion method

Tail immersion test was performed according to procedure as described by Olaleye SB et al. [32]. The mice tail (1 to 2 cm) was immersed in warm water kept constant at 55 ± 1 °C. The reaction time means is the time when mice deflect their tails. The first reading was discarded and the reaction time was calculated as a mean of the next three consecutive readings that was recorded at an interval of 24 h. A latency period of 28 s was distinct as complete analgesia and the evaluation was then stopped to keep away from injury of mice. The latent period of the tail-immersion response was counted at 0, 30, 60 and 90 min after the administration of standard and test drugs. Elongation percentage was calculated using the following formula

$$ \mathrm{Elongation}\%=\left\{\left(\mathrm{Latency}\  \mathrm{of}\  \mathrm{test}\  \mathrm{animal}\right)\hbox{--} \left(\mathrm{Latency}\  \mathrm{of}\  \mathrm{control}\  \mathrm{animal}\right)\right\}/\left(\mathrm{Latency}\  \mathrm{of}\  \mathrm{control}\  \mathrm{animal}\right)\times 100 $$

### Determination of anti - inflammatory activity by carrageenan-induced paw oedema method

The six groups (each containing 5 mice) were taken for the test. The injected 0.1 mL carrageenan (1%) into plantar surface of mice hind paw can create acute inflammation [31]. After 30 min of carageenan injection, the treated animals received MEBRP and MEBRS (100 and 200 mg/kg, p.o), respectively. Tween 80 and Ibuprofen, (10 mg/kg, p.o.), were given in negative and positive control, respectively. The paw volume was measured at 1 h, 2 h, 3 h, and 4 h using a vernier caliper to determine the diameter of oedema.

### Determination of CNS depressant activity by hole cross test

The method included a specific type of cage which consists of a steel partition that fixed in the middle of a cage having a size of 30 × 20 × 14 cm. In the center of the cage, a hole of 3 cm diameter was made at a height of 7.5 cm [33]. Animals were divided into four groups (*n* = 5) and each group containing four mice. Control mice received vehicle (1% Tween 80 in water), positive control received diazepam (1 mg/kg body weight, p.o.); the treated animals received MEBRP (100 and 200 mg/kg, p.o) and MEBRS (100 and 200 mg/kg, p.o), respectively. After oral administration of test drugs, the number of mice passages through the hole from one chamber to other was calculated for a period of 3 min at 0, 30, 60, 90 and 120 min.

### Determination of CNS depressant activity by open field test

The experiment was carried out according to the methods described by [34]. The floor of an open field divided into alternatively colored black and white squares and the wall height was 40 cm. After giving test drugs, the number of animal movements was counted up to 3 min at 0, 30, 60, 90 and 120 min.

### Determination of anti-diarrheal activity by castor oil induced diarrhea

This study was conducted by the method explained by Shoba and Thomas [35]. Initially 0.5 mL castor oil is given to each mouse for screening and only mice those showing diarrhea were chosen for the final experiment. The animals were divided into following six groups containing five mice. Control was treated with vehicle (saline 10 mL/kg, p.o.); the treated mice received MEBRP (100 and 200 mg/kg, p.o) and MEBRS (100 and 200 mg/kg, p.o), respectively. Positive control received loperamide (3 mg/kg body weight, p.o). The blotting paper was previously placed in each case and then animal was kept in an individual cage. The floor lining was changed every hour. After 30 min diarrhea was induced by oral administration of 0.5 mL castor oil. The total number of fecal output and the number of diarrheic feces excreted by the animals were recorded up to 4 h.

### Statistical analysis

All values were expressed as the means ± S.E.M. of five mice (*n* = 5). The data were analyzed by ANOVA (Analysis of variance) followed by Dunnett’s test (Statistical Package for Social Sciences, SPSS 16.0, USA). *P* values < 0.05 was considered as significant.

## Results

### Brine shrimp lethality bioassay

In this test the LC_50_ value of MEBRP 40 μg/mL, LC_50_ value of MEBRS 10 μg/mL where LC_50_ of standard (vincristine sulphate) was 0.83 μg/mL. The Brine Shrimps lethality was found to be concentration-dependent (Table 1).

**Table 1:** Brine Shrimp lethality bioassay of themethanolextract of the *Baccaurea ramiflora* pulp and seed

Concentration (μg/mL)	Log C	No. of dead shrimps (out of10)		% of mortality		LC_50_ (μg/mL)		
		MEBRP	MEBRS	MEBRP	MEBRS	MEBRP	MEBRS	Vincristine sulphate
10	1	3	5	30	50	40 ± 1.05	10 ± 0.63	0.83 ± 0.25
20	2.08	4	6	40	60			
40	0.32	5	6	50	60			
80	1.90	6	7	60	70			
160	2.20	6	7	60	70			

Values are means ± S.E.M. (standard error means) of three independent experiments. *MEBRP* Methanol Extract of *Baccaurea ramiflora* pulp*, MERBS* Methanol Extract of *Baccaurea ramiflora* seed

### Analgesic activity

#### Acetic acid induced writhing in mice

**Fig. 1:**
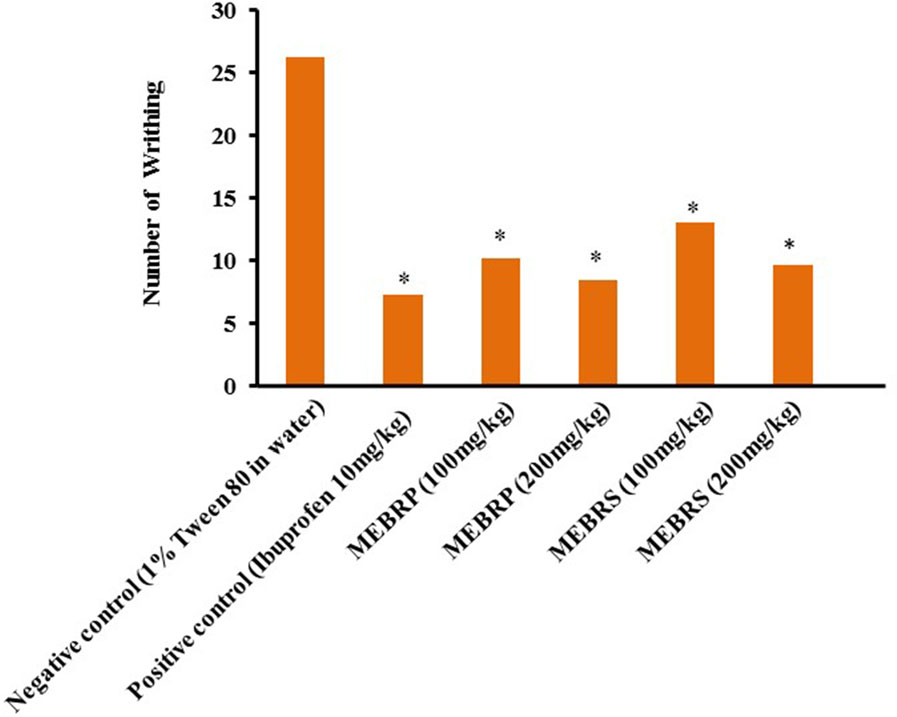
Analgesic effects of methanol extract of the *Baccaurea ramiflora* pulp and seed on acetic acid-induced writhing in mice. Values are means ± SEM of five mice (*n* = 5); *p < 0.05 as compared to control (One way ANOVA followed by Dunnett’s test). Control mice received vehicle (1% Tween 80 in water), positive control received Ibuprofen 10 mg/kg body weight, tested animals were treated with 100 and 200 mg/kg body weight (p.o.) of the MEBRP and MEBRS, respectively

In the acetic acid induced writhing method the MEBRP, MEBRS (200 mg/kg) showed almost same % of inhibition (67.51 and 66.08%, respectively) compared to standard (72%) and in a dose dependent manner (Fig. 1).

#### Formalin induced licking and biting test

**Fig. 2:**
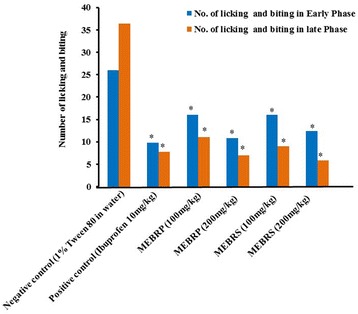
Analgesic effects of the methanol extract of *Baccaurea ramiflora* pulp and seed on hind paw licking in the formalin test in mice. Values are means ± SEM of five mice (*n* = 5); *p < 0.05 as compared to vehicle control (One way ANOVA followed by Dunnett’s test). Control mice received vehicle (1% Tween 80 in water); positive control received Ibuprofen 10 mg/kg body weight; tested animals were treated with 100 and 200 mg/kg body weight (p.o.) of the MEBRP and MEBRS, respectively

The MEBRP, MEBRS (200 mg/kg) have shown 58.5 and 53.4% protections respectively in the early phase but in the late phase % of protections of MEBRP, MEBRS were 80.8 and 76.61% respectively where standard was 62.30 and 78.6% protection in the early and late phase respectively (Fig. 2).

#### Tail immersion test

**Fig. 3:**
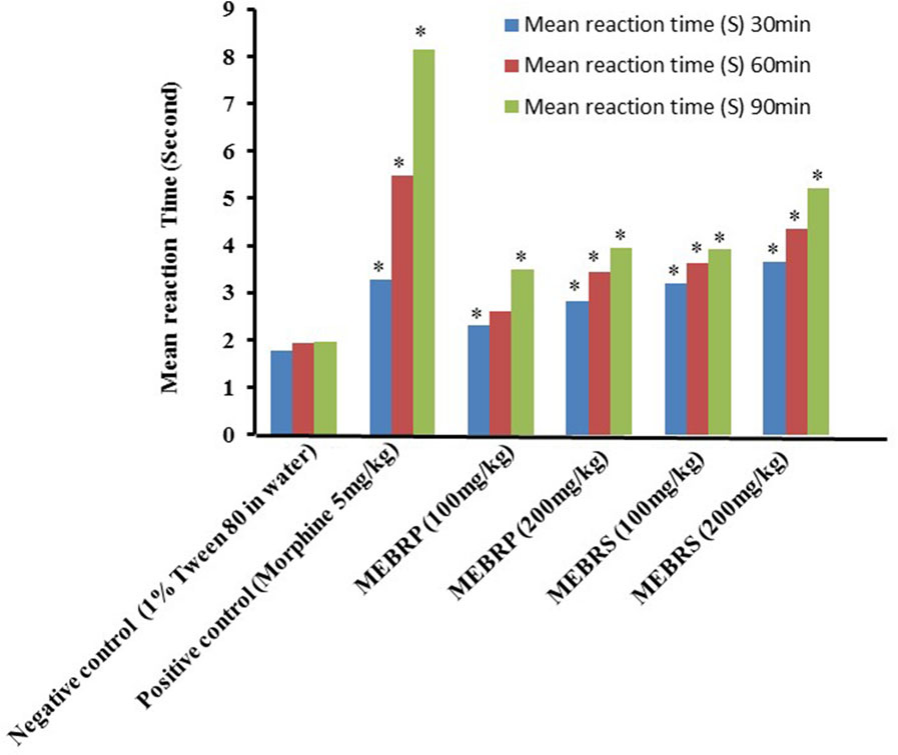
Analgesic effects of the methanol extract of the *Baccaurea ramiflora* pulp and seed on tail immersion test in mice. Values are means ± SEM of five mice (*n* = 5); * *p <* 0.05, Dunnett’s test compared to control. Control group received normal saline; standard groups received morphine 5 mg/kg body weight (i.p.); test animals were treated with 100 and 200 mg/kg (p.o.) body weight of the MEBRP and MEBRS, respectively

The maximum effect was observed at 60 and 90 min of drug administration in tail immersion test. The dose dependent 50.16%, 62.4% thermal stimulus inhibitions have shown by the MEBRP and MEBRS (200 mg/kg), respectively. In this study morphine (75.9% inhibition) was used as standard (Fig. 3).

### Carrageenan induced paw edema test

The MEBRP, MEBRS (200 mg/kg) exhibited moderate anti-inflammatory activity (42 and 55.22% inhibitions, respectively) and the % of inhibition of the standard (ibuprofen) was 74.62%. The anti-inflammatory activities of both pulp and seed extract were dose dependent (Table 2).

**Table 2:** Anti-inflammatory effects of the methanol extract of the *Baccaurea ramiflora* pulp and seed on carrageenan induced paw edema in mice

Group	Dose	Oedema diameter (mm)				Inhibition (%)			
		1 h	2 h	3 h	4 h	1 h	2 h	3 h	4 h
Negative Control (1% tween 80 in water)	–	4.66 ± 0.84	4.36 ± 0.66	4.28 ± 0.40	4.02 ± 0.43	–	–	–	–
Positive control (Ibuprofen)	10 mg/kg	2.46 ± 0.42^*^	1.92 ± 0.44^*^	1.44 ± 0.39^*^	1.02 ± 0.36^*^	47.21	56	66.4	74.62
MEBRP	100 mg/kg	3.64 ± 0.51^*^	3.44 ± 0.44^*^	3.08 ± 0.52^*^	2.76 ± 0.46^*^	21.9	21.10	28.03	31.34
MEBRP	200 mg/kg	3.28 ± 0.41^*^	3.12 ± 0.50^*^	2.74 ± 0.57^*^	2.34 ± 0.49^*^	29.61	28.44	36	42
MEBRS	100 mg/kg	3.8 ± 0.42^*^	3.4 ± 0.50^*^	2.96 ± 0.23^*^	2.38 ± 0.48^*^	18.5	22.08	30.84	40.80
MEBRS	200 mg/kg	3.44 ± 0.40^*^	2.82 ± 0.38^*^	2.34 ± 0.49^*^	1.8 ± 0.27^*^	26.18	35.32	45.33	55.22

Values are means ± S.E.M. of five mice (*n* = 5)

^*^p < 0.05 considered as significant compared to control (One way ANOVA followed by Dunnett’s test). Control mice received vehicle (1% Tween 80 in water); positive control group received Ibuprofen 10 mg/kg body weight; MEBRP and MEBRS treated groups were administered with 100 and 200 mg/kg body weight (p.o.), respectively

### CNS depressant activity

#### Hole-cross test

MEBRP, MEBRS (200 mg/kg) showed high sedative activity at 30, 60, 90, and 120 min. Both pulp and seed have dose dependent activity and they all were statistically significant (*p* < 0.05) (Table 3).

**Table 3:** Depressant effects of the methanol extract of the *Baccaurea ramiflora* pulp and seed on hole cross test in mice

Group	Dose	Number of Movements				
		0 min	30 min	60 min	90 min	120 min
Negative Control (1% tween 80 in water)	–	9.0 ± 1.26	7.2 ± 1.28	7.6 ± 1.35	9.0 ± 1.26	10 ± 1.26
Positive control (Diazepam)	1 mg/kg,	6.6 ± 1.07^*^	5.4 ± 1.07^*^	3.8 ± 0.92^*^	3.8 ± 1.47^*^	3 ± 1.26
MEBRP	100 mg/kg	5.2 ± 0.92^*^	5.0 ± 0.84^*^	4.8 ± 0.92^*^	4.6 ± 0.95^*^	3.8 ± 0.92^*^
MEBRP	200 mg/kg	4.8 ± 0.92^*^	4.6 ± 0.74^*^	3.4 ± 0.74^*^	3.0 ± 0.84^*^	2.6 ± 0.74^*^
MEBRS	100 mg/kg	5.2 ± 1.14^*^	4.2 ± 1.14^*^	4.2 ± 1.14^*^	2.6 ± 1.07^*^	2 ± 0.84^*^
MEBRS	200 mg/kg	4.8 ± 0.92^*^	4.0 ± 0.84^*^	2.8 ± 0.92^*^	1.8 ± 0.92^*^	1 ± 0.84^*^

Values are means ± S.E.M. of five mice (*n* = 5)

^*^p < 0.05 considered as significant compared to control (Dunett’s test). Control mice received vehicle (1% Tween 80 in water), positive control mice received diazepam 1 mg/kg body weight, MEBRP and MEBRS treated groups were administered with 100 and 200 mg/kg body weight (p.o.), respectively

#### Open-field test

In the open field test MEBRP, MEBRS (200 mg/kg) showed same sedative activity at 60, 90, and 120 min compared to standard. The sedative effects of both pulp and seed were dose dependent (Table 4).

**Table 4:** Depressant effects of the methanol extract of the *Baccaurea ramiflora* pulp and seed on open field test in mice

Group	Dose	Number of Movements				
		0 min	30 min	60 min	90 min	120 min
Negative Control (1% tween 80 in water)	–	239.40 ± 2.60	189.6 ± 7.01	176.8 ± 2.42	159 ± 2.72	146 ± 2.34
Positive control (Diazepam)	1 mg/kg,	89 ± 1.84^*^	87.4 ± 1.347^*^	70 ± 1.92^*^	65 ± 1.78^*^	51 ± 1.88^*^
MEBRP	100 mg/kg	201 ± 2.62^*^	158 ± 3.22	76 ± 2.55^*^	68 ± 2.90^*^	51.6 ± 2.72^*^
MEBRP	200 mg/kg	180.8 ± 2.614^*^	148.2 ± 3.64^*^	66 ± 2.04^*^	52 ± 3.21^*^	42 ± 2.39^*^
MEBRS	100 mg/kg	206.8 ± 2.314^*^	170 ± 2.81	79 ± 2.454^*^	72.8 ± 3.05^*^	52.2 ± 2.95^*^
MEBRS	200 mg/kg	169 ± 3.783^*^	120 ± 5.05^*^	70 ± 4.742^*^	61 ± 3.55^*^	49.6 ± 2.04^*^

Values are means ± S.E.M. of five mice (*n* = 5)

^*^p < 0.05 considered as significant compared to control (Dunett’s test). Control animals received vehicle (1% Tween 80 in water), positive control mice received diazepam 1 mg/kg body weight, MEBRP and MEBRS treated groups were administered with 100 and 200 mg/kg body weight (p.o.), respectively

### Anti-diarrhoeal activity

The MEBRP, MEBRS (200 mg/kg) decreased the number of diarrhea (castor oil induced) of the test animals and the % inhibitions for defecations were 59.7 and 63.03%, respectively compared to standard (loperamide 61.34%) (Table 5).

**Table 5:** Antidiarrheal effects of the methanol extract of the *Baccaurea ramiflora* pulp and seed on castor oil induced diarrhea in mice

Group	Dose	No. of faces in 4 h	% inhibition of defecation
Negative Control (1% tween 80 in water)	–	23.8 ± 1.6	–
Positive control (loperamide)	3 mg/kg	9.2 ± 1.14^*^	61.34
MEBRP	100 mg/kg	12 ± 1.6^*^	50
MEBRP	200 mg/kg	9.6 ± 1.1^*^	59.7
MEBRS	100 mg/kg	11.6 ± 1^*^	51.26
MEBRS	200 mg/kg	8.8 ± 1.1^*^	63.03

Values are means ± S.E.M. of five mice (*n* = 5)

^*^p < 0.05 considered as significant compared to control (One-way ANNOVA followed by Dunnet’s test). Control mice received vehicle (1% Tween 80 in water), positive control mice received loperamide 3 mg/kg body weight, MEBRP and MEBRS treated groups were administered with 100 and 200 mg/kg body weight (p.o.), respectively

## Discussion

The diverse pharmacologic actions, cytotoxic, and pesticidal effects can be identified by the easy brine shrimp test [36]. The active plant compounds are responsible for biological responses. The brine shrimp method can determine the biological activities of natural products [37]. The *B. ramiflora* has brine shrimp’s mortality activity [38]. The MEBRP, MEBRS have less cytotoxic effect compared to vincristine sulphate. Moreover it is also noticeable that theLC_50_ value of MEBRP is higher compared toLC_50_ value of MEBRS. The writhing method involved peripherally acting analgesic and represents pain sensation by triggering localized inflammatory response which stimulates tissue phospholipid to release free arachidonic acid [39]. These reactions can be regulated by the prostaglandin pathways [40], peritoneal mast cells [41], and acid sensing ion channels [42].

Generally inflammatory pain is reduced by non-steroidal anti-inflammatory and analgesic drugs which can inhibit the production of pain mediators which are initiated by prostaglandins and bradykinin [43]. Therefore, the *B. ramiflora* might have peripheral analgesic action that influences the local reaction which is caused by the various types of production, secretion or antagonizing the action of pain mediators at the target sites. The MEBRP, MEBRS (200 mg/kg body weight) have almost same analgesic activity against pain which is caused by acetic acid in mice and these analgesic activities were very close to the reference drug (Ibuprofen). These results indicate that the *B. ramiflora* pulp and seed are related to the peripheral mechanisms in the analgesic action. Pritam et al. proved that compounds like flavonoids, steroids and triterpenes have anti-inflammatory and anti-nociceptive activities [44].

The formalin model is represented by neurogenic phase (0–5 min) and inflammatory pain phase (15–30 min) respectively [45]. The drugs (e.g. steroids or NSAID) primarily suppress the late phase in the CNS [46]. The neurogenic and inflammatory pains reduction by the extract might indicate the presence of analgesic compound which may work peripherally or centrally. As a result this extract can remove chronic as well as acute pain. In recent time, McNamara et al. [47] showed that formalin stimulates primary afferent neurons through a definite TRPA1 which is related to the cation channels, a subset of C-fiber nociceptors. This outcome is regulated by increased influx of Ca2+ ions. Moreover, TRPA1 cation channels mediate noxious mechanical stimuli [48]. These investigations suggested that the TRPA1 related Ca^2+^ mobilization is connected with mechanical stimuli and noxious chemicals since analgesic action is produced. Similarly, the inhibitory pain response of *B. ramiflora* might be prevent of the increase intracellular Ca^2+^induced by formalin. The MEBRP, MEBRS (200 mg/kg) showed significant analgesic action at both early phases and at late phase. Like acetic acid induced analgesic method formalin model also focused that both MEBRP and MEBRS (200 mg/kg) have same analgesic activity compared to standard drug and all data were statistically significant (*p* < 0.05).

The MEBRP, MEBRS extract (200 mg/kg, p.o.) inhibited neurogenic pain at both first and second phase induced by formalin. Thus the results indicate *B. ramiflora* possesses significant prostanoid mediators of inflammation [49]. The acute pain of tail immersion response is predominantly a measure for centrally acting analgesics. But in case of heat-induced pain drugs acting peripherally are inactive [50]. Latency time of mice to thermal stimuli is calculated in the tail immersion test. The different compounds in the extracts that cause calcium ions influx at the afferent nerve can also reduce adenylcyclase activity. Resulting reactions reduce cAMP level and cause an efflux of potassium ions. The resulting hyperpolarization leads to analgesic effects [51]. The tail-withdrawal time significantly (*p* < 0.05) increased at 30, 60 and 90 min indicate that MEBRP, MEBRS have centrally acting analgesic activity. Since the MEBRP and MEBRS (200 mg/kg) have moderate inhibition which specify the central analgesic effects of MEBRP, MEBRS. Therefore, the resulting analgesic data of three models indicate that the presence of pharmacologically active phytoconstituent (centrally and peripherally) in the MEBRP, MEBRS (200 mg/kg) extracts.

Carrageenan stimulate the release of proinflammatory and inflammatory mediators like bradykinin, leukotrienes prostaglandins, TNF-α, histamine etc. [52]. Moreover during tissue damage and inflammation neutrophils are stimulated to release excessive NO and ROS which are responsible for a variety of disease [53]. In case of experimental animal model, carrageenan is used for acute inflammation which is a biphasic. Initially, serotonin, histamine and increased synthesis of prostaglandins in the damaged tissue surroundings are involved in the first phase. Afterward, the second phase is continued by bradykinin, prostaglandins, polymorphonuclear cells, leukotrienes etc. [54, 55]. The methanol extract MEBRP, MEBRS were evaluated for in vivo analgesic and anti-inflammatory properties. Since the MEBRP, MEBRS (200 mg/kg) significantly inhibited paw edema in the second phase. This indicates inhibition of cyclooxygenase by the *B. ramiflora* extracts. In this test the effects were moderate compared to standard (Ibuprofen), which also inhibit the cyclooxygenase enzyme action. The pain perceptions as well as inflammations are inhibited by flavonoids and saponins which has inhibitory effects to the formation of inflammatory mediators. These results suggest that *B. ramiflora* may be act as an anti-inflammatory compound.

Increase of locomotor activity indicates alertness whereas decrease of locomotor activity indicates sedative effect [56]. Gamma-aminobutyric acid (GABA) is the major inhibitory neurotransmitter. Different drugs such as sedative-hypnotic, anxiolytic, muscle relaxant revealed their activity through GABA, therefore the methanol extracts *B. ramiflora* can act through enhancing GABAergic inhibition in the CNS that causes a reduction in the firing rate of critical neurons or direct activation [53] of GABA receptor. During study period of hole cross method, the MEBRP, MEBRS (100 and 200 mg/kg) have shown significant depressant activity at 30, 60, 90, 120 min. Besides this, at 30, 60, 90 and 120 min, the counted squares of each animal were also significantly decreased. In a previous study many flavonoids and neuroactive steroids may act as ligands for the GABA receptors in the central nervous system. This activity is similar to benzodiazepine like molecules [56].

The castor oil induced diarrheal effect has a number of mechanisms such as adenylatecyclase or mucosal cAMP mediated active secretion [57], inhibition of intestinal Na+, K + -ATPase activity to lessen normal fluid absorption [58], platelet activating factor and nitric oxide have contribution to the diarrheal effect [59] and prompting of prostaglandin production [60] etc.

The castor oil containing nitric oxide stimulate diarrheal activity, as well as the ricinoleic acid also created diarrhea through a hypersecretory response which is the most active chemical of castor oil [61]. In this test, the number of the feces of the test animals decreases within 4 h. The MEBRP, MEBRS (100 mg/kg) showed moderate inhibitory activity against defecation; whereas 200 mg/kg dose inhibition was close to the standard drug loperamide. Hence, these results indicate the pulp and seed extracts might have antidiarrheal action.

## Conclusions

Our study demonstrates the effectiveness of *Baccaurea ramiflora* pulp and seed (200 mg/kg) extracts for analgesic, anti-inflammatory, CNS depressant and anti-diarrheal activities. Thus, the extracts are expected to contain active ingredient (s) that may contribute for the isolation of new bioactive compound (s). Hence, we suggest for further studies on the isolation and evaluation of isolated compounds in vitro and in vivo.

## Abbreviations

*B. ramiflora*: *Baccaurea ramiflora*
MEBRP: Methanol extracts of *Baccaurea ramiflora* pulp
MEBRS: Methanol extracts of *Baccaurea ramiflora* seeds
